# Role of Surface Morphology Evolution in the Tribological Behavior of Superalloy Under High-Temperature Fretting

**DOI:** 10.3390/ma18102350

**Published:** 2025-05-18

**Authors:** Xuan He, Zidan Wang, Ying Yan, Kailun Zheng, Qian Bai

**Affiliations:** State Key Laboratory of High-Performance Precision Manufacturing, Dalian University of Technology, Dalian 116024, China; hexuan823@163.com (X.H.); wangzidan21@mail.dlut.edu.cn (Z.W.); zhengkailun@dlut.edu.cn (K.Z.)

**Keywords:** superalloy, surface morphology evolution, fretting wear, wear mechanism

## Abstract

High-temperature fretting wear typically occurs on mechanical contact surfaces in high-temperature environments, with displacement amplitudes generally in the micrometer range (≤300 μm), such as the turbine disks and blades in aerospace engines, and the piston rings in automotive engines. The study performed tangential fretting wear tests between superalloy specimens and Si_3_N_4_ balls under 700 °C to investigate the influence of ground and milled surface morphologies on the high-temperature fretting wear behavior. The experimental results show distinct wear mechanisms for the two surface types: ground specimens exhibit adhesive and oxidative wear, while milled specimens experience fatigue and abrasive wear. Both wear modes intensify with increasing load and fretting frequency. A comprehensive surface morphology characterization method, combining fractal dimension (*FD*) and surface roughness, is proposed. The study reveals that the roughness parameters *Sa* and *Ra* are strongly correlated with the Coefficient of Friction, while *FD* is strongly correlated with the wear volume. This study provides a novel approach to characterizing the evolution of surface morphology during high-temperature fretting wear.

## 1. Introduction

High-temperature fretting wear typically occurs on mechanical contact surfaces in high-temperature environments, particularly in areas subjected to alternating loads or cyclic motion, such as the turbine disks and turbine blades in aerospace engines, and the piston rings in automotive engines. Superalloy exhibits excellent high-temperature mechanical properties, high-temperature oxidation resistance, and high-temperature thermal stability. It is capable of operating for extended periods under complex stress conditions at temperatures above 600 °C [1,2]. Therefore, it is widely used in mechanical components in the aerospace industry [3], shrink-fit connections [4], automotive, instrumentation, and medical devices [5]. In aero-engines, superalloy turbine disks are typically connected to turbine blades via dovetail joints. Under the severe conditions of high temperature, high pressure, and high rotational speed, periodic micrometer-scale fretting wear occurs at the contact surfaces between the joint surface of the turbine disk and the turbine blade [6]. This wear can easily lead to the initiation and propagation of fatigue cracks in the dovetail joints [7], resulting in reduced fatigue life or even fracture, which severely affects the service life and reliability of aero-engines [8]. Therefore, investigating the tribological properties of superalloys under high-temperature fretting wear conditions is of great significance for improving the lifespan of key components.

The fretting wear behavior is primarily influenced by factors such as load, frequency, and temperature. Hu et al. [9] showed that IN738LC alloy underwent thermoplastic deformation under the combined action of normal load and tangential friction force, which resulted in the accumulation of strong dislocation densities and diverse deformed tissue in damage zones. Zurcher et al. [10] studied and showed that under the conditions of 20 N and 400 °C, a glaze layer composed of oxidized fragments was formed on the wear surface of IN718 alloy, while at 50 N, the oxidized fragments did not adhere to the contact area, and the abrasive wear was more severe. Hu et al. [11] showed that as the load increases, the micro motion state of IN738LC alloy transitions from the gross slip regime to the mixed slip regime, and both the wear volume and wear rate decrease. Jeong et al. [12] showed that the micro motion wear mechanism of Inconel 690 alloy in air environment is delamination wear, and the wear volume decreases with increasing load. He et al. [13] studied that the third body layer of GH2132 alloy reduces the CoF and fluctuations in the wear zone, thus reducing the degree of wear.

Turbine disk dovetail joints and other components in aero-engines are typically manufactured using ground or milled processes. Different processing conditions result in distinct surface morphologies, which significantly influence tribological properties [14]. Wang et al. [15] showed that the Inconel 25 alloy with the highest roughness exhibited the lowest CoF and wear rate, indicating the best high-temperature wear resistance. Under high-temperature fretting conditions, the surface morphology of the specimen is continuously altered, which in turn may affect the tribological properties. However, current research has primarily focused on the influence of parameters such as load, amplitude, and frequency on fretting characteristics, while investigations into the effects of surface morphology evolution on fretting behavior remain insufficient. This knowledge gap substantially hinders accurate lifespan prediction of components under high-temperature fretting conditions.

## 2. Experimental Details

### 2.1. Materials and Preparations

**Figure 1:**
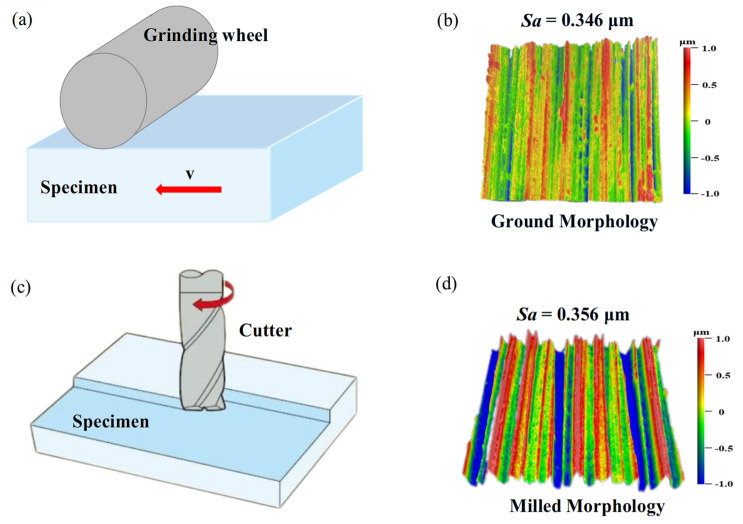
(**a**) Ground processing; (**b**) ground morphology; (**c**) milled processing; (**d**) milled morphology.

In this study, specimens of FGH96 were used—the main chemical composition is shown in Table 1—and the material properties are shown in Table 2. The material was machined into rectangular specimens with dimensions of 35 mm × 25 mm × 9 mm. One surface (35 mm × 25 mm) of each specimen was subjected to ground and milled processes. The ground process was performed at a cutting speed of 2800 r/min with a depth of cut of 0.02 mm. The milled process was conducted with a feed rate of 0.1 mm/z, a cutting speed of 800 r/min, and a depth of cut of 0.2 mm. The schematic diagram of the milled process and the surface morphology after machining are shown in Figure 1. The surface roughness values of the ground and milled surfaces were 0.346 μm and 0.356 μm, respectively. The standard [16] specifies that the diameter of the ball specimen in friction and wear tests should range from 2 to 10 mm. Additionally, the UMT-500 friction and wear testing machine recommends using a ball with a diameter of 9.525 mm [17]. Therefore, we selected the Si_3_N_4_ friction ball with a diameter of 9.525 mm and a surface roughness of less than 0.05 μm.

### 2.2. Tribological Test

**Figure 2:**
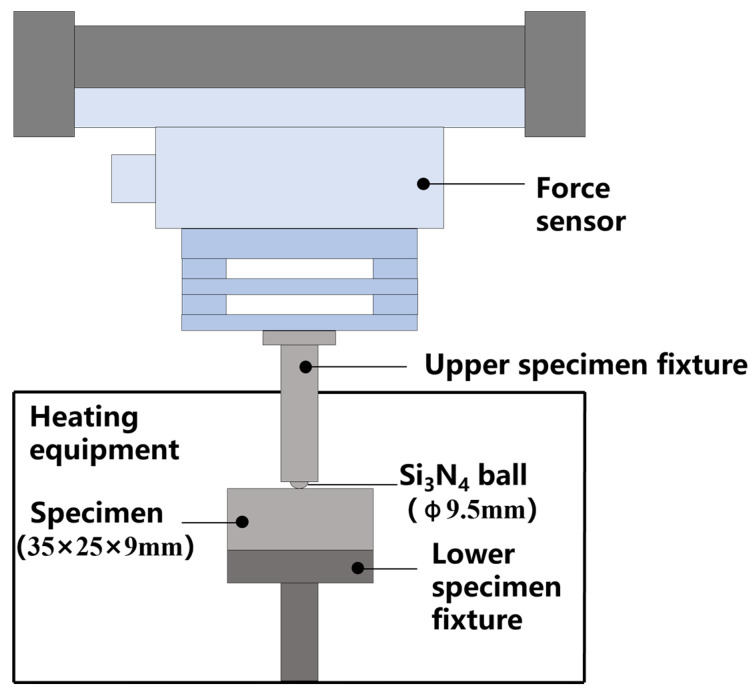
Schematic diagram of high-temperature fretting test.

The tribological tests were conducted on the rotary module of a tribometer (UMT-500, Bruker, Billerica, MA, USA). The friction tests were performed with load and frequency as experimental variables to investigate the friction and wear mechanisms of FGH96 with different surface morphologies. The FGH96 specimen was mounted on the fixture of the test disk, while the Si_3_N_4_ ball was installed in the pin fixture. The experimental setup is shown in Figure 2. In order to investigate the fretting wear characteristics of FGH96 in a wider range of operation conditions, four loads, i.e., 50 N, 100 N, 150 N, and 200 N, were employed. To enhance test efficiency, a test time of 1800 s was used, which is sufficient to study the influence of surface morphology evolution on the fretting behavior. All tests were conducted at a temperature of 700 °C with a fretting amplitude of 300 μm. For the frequency variation tests, the FGH96 specimen was subjected to a load of 150 N at different frequencies. In order to study the surface morphology evolution, tests were conducted at 20 Hz and 150 N by varying the wear duration. The detailed test conditions are described in Table 3.

All tests were repeated three times, and the average value of CoF was calculated. In this study, the wear volume was calculated based on the measured results from the optical profilometer (ZYGO AMETEK, Middlefield, CT, USA). The data of 3D topography for the wear scar were acquired by using a phase-shifting interferometry scanning mode. A reference plane of the unworn area was established, and the wear scar boundary was defined by using the volume parameter function. Then, the wear volume was calculated after applying Gaussian filtering (λc = 0.8 mm). To ensure data reliability, three measurements at each condition are employed.

### 2.3. Box-Counting Method for Calculating Fractal Dimension

The wear surface morphology refers to the micro-surface morphology formed by the interaction of components in a friction pair during the friction and wear process [18,19,20]. The wear surface morphology can be reflected by two-dimensional surface profiles and three-dimensional surface morphology. The two-dimensional surface profile is the intersection line between the three-dimensional surface morphology and a vertical cross section in any direction. The wear surface profile is commonly characterized by roughness parameters, such as the arithmetic mean deviation (*Ra*) and the arithmetic mean height (*Sa*). The friction system is a complex nonlinear dynamical system, and the wear surface also exhibits nonlinearity. Fractals are phenomena characterized by self-similarity and self-affinity, lacking a characteristic scale and containing elements of all scales. Their complex details can be observed at every scale, allowing for the description of the complexity of curves and surfaces [21]. These advantages make fractal theory an important method for characterizing wear surface morphology [22,23].

Therefore, in this study, fractal dimension (*FD*) is used to characterize the wear surface. We use the box-counting method (BCM) to calculate the *FD*, as shown in Equation (1) [24]:

(1) $$FD=\underset{\varepsilon \to 0}{\lim}\frac{ln(N_{\varepsilon })}{ln1/\varepsilon }$$

In the formula, *ε* represents the box size, and *Nε* is the minimum number of boxes.

**Figure 3:**
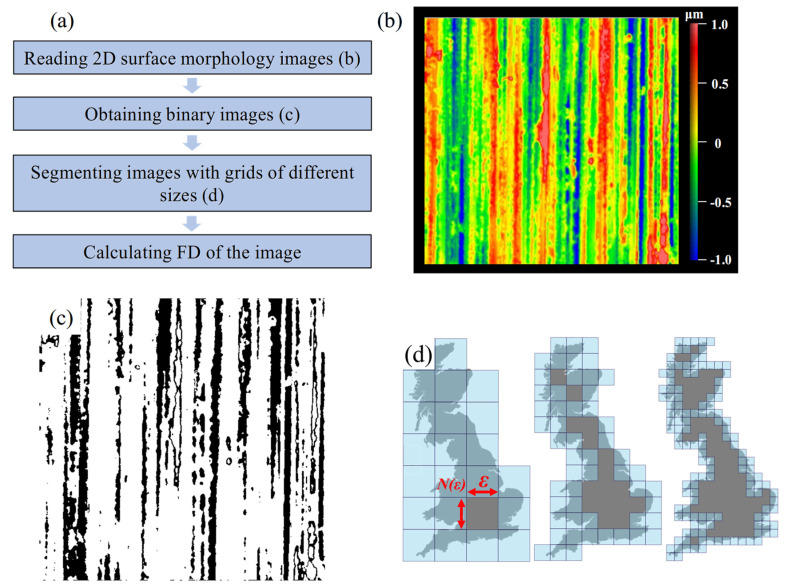
(**a**) The steps for BCM to calculate FD; (**b**) standardization of the original surface of the ground specimen; (**c**) binary image; (**d**) BCM schematic diagram.

The steps for calculating the fractal dimension (*FD*) are illustrated in Figure 3a. First, normalize the color bar range between 1 μm and −1 μm (Figure 3b). Then, obtain the binary image of each specimen (Figure 3c). The box size *ε* is successively taken as 2*^−p^* (*p* = 1, 2…) in sequence, as shown in Figure 3d.

**Figure 4:**
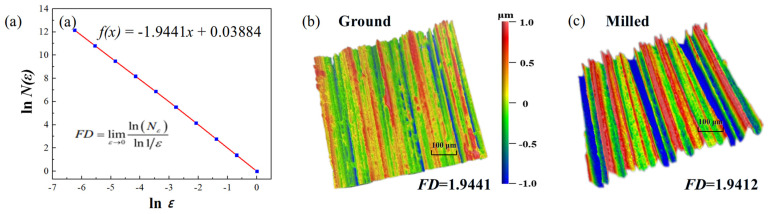
(**a**) Calculation of *FD*; 3D surface morphologies for original surface of FGH96: (**b**) ground specimens; (**c**) milled specimens.

Use linear regression analysis to calculate the fractal dimension (*FD*) of the ground and milled specimens in the initial state (Figure 4a). According to Equation (1), the absolute value of the slope of the curve is 1.9441, which represents the *FD* value for the ground specimen. Similarly, the *FD* value for the milled specimen in its initial state is 1.9412.

## 3. Results and Discussion

### 3.1. Effect of Surface Morphology on Tribology Characteristics

#### 3.1.1. Effect of Surface Morphology on CoF

**Figure 5:**
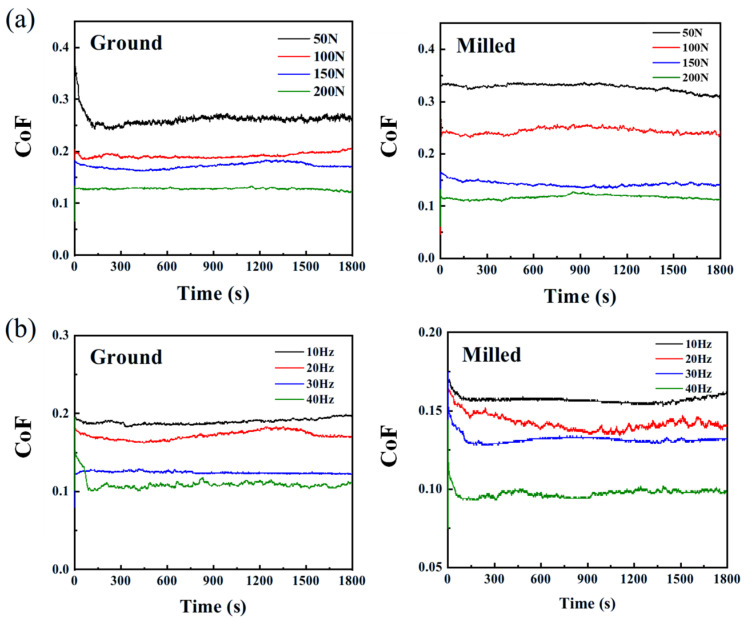
Variation curves of CoF for ground and milled specimens under different (**a**) loads and (**b**) frequencies.

In the fretting wear process, the CoF is an important parameter, as it can reflect the wear degree of the specimen at different stages during wear [25]. All the CoF values throughout the wear process in our analysis is considered [26]. Figure 5 presents the variation curves of the CoF for ground and milled specimens under different loads and frequencies. The CoF in the wear zones of both ground and milled specimens decreases with increasing load (Figure 5a). This is because under plastic deformation, the growth rate of the actual contact area lags behind the increase in load, leading to stabilized contact stress. The surface asperities form flatter contact interfaces, thereby reducing stress concentration. Moreover, higher loads accelerate the oxidation and grinding of wear debris, and an oxide film composed of various oxides such as Al_2_O_3_, TiO_2_, Cr_2_O_3_, and NiO covers the contact interface and acts as a solid lubricant [27]. Consequently, the CoF decreases with the increasing load. The results demonstrate that the wear processes all reached dynamic steady state within 500 s, thus 500 s as the test duration is enough.

As shown in Figure 5b, when the frequency increases from 10 Hz to 40 Hz, the CoF in the wear zones of both ground and milled specimens decreases with increasing frequency. This is because, as the frequency increases, the number of wear cycles per unit time rises, leading to an increase in the temperature of the contact surfaces of the friction pair. Under the influence of high temperature, oxidation reactions are the main wear mechanism, and a friction oxide film gradually forms on the alloy surface. The isolation and protection effect of this oxide film reduces the CoF. This results in the continuous shedding of wear debris, which act as abrasive particles and scrape the surface, thus the CoF value increases. Additionally, higher frequencies lead to increased temperatures, resulting in the plastic deformation of asperities, thereby intensifying the wear and the CoF value increases further.

#### 3.1.2. Effect of Surface Morphology on Wear Volume

**Figure 6:**
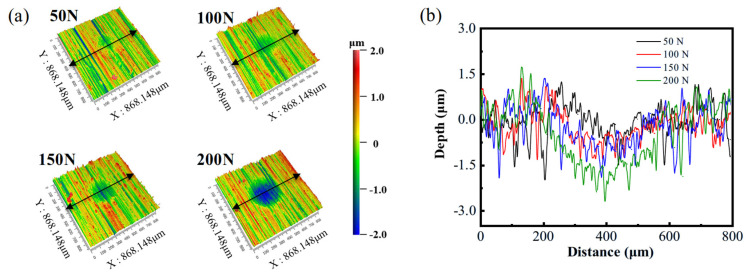
(**a**) Three-dimensional morphology and (**b**) two-dimensional contours of the observed contact zone.

The three-dimensional morphologies of the wear zones on ground specimens after high-temperature fretting wear were obtained using a 3D interferometer (ZYGO, AMETEK, Middlefield, CT, USA) (Figure 6a). The corresponding two-dimensional profiles along the cross line as shown in Figure 6a are shown in Figure 6b. The results indicate that as the load and frequency increase, the wear surface becomes more pronounced, and the wear scars deepen. This is attributed to the larger contact area between the upper and lower specimens under higher loads, which leads to an increase in the number of asperity contacts. Consequently, the actual contact area grows with the increasing load. Since the frictional resistance is positively correlated with the actual contact area, the wear intensifies with higher loads. The increasing frequency accelerates the peeling of materials from the wear zone, and abrasive wear dominates the main wear mechanism. Therefore, the wear amount increases with the increasing frequency.

**Figure 7:**
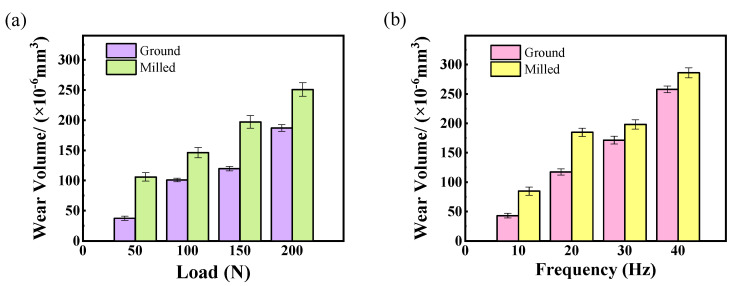
Wear volume of ground and milled specimens under different experimental conditions: (**a**) loads; (**b**) frequencies.

Figure 7 presents the variation trends of wear volume in the wear zones of ground and milled specimens under different load and frequency conditions, respectively. As shown in Figure 7a, the wear volume in the wear zones of both ground and milled specimens increases with increasing load. Under higher loads, the generated abrasive particles cause uneven stress distribution on the surface of the superalloy, leading to stress concentration and promoting the formation of surface microcracks. As wear progresses, these cracks detach from the surface in contact with the debris, resulting in an increase in wear volume [28].

Figure 7b indicates that the wear volume in the wear zones of both ground and milled specimens increases with increasing frequency. The small spread of wear values may be due to the high load during the fretting test. At the initial stage of wear, only a few asperities on the upper and lower specimens are in contact, resulting in a very small actual contact area. As wear continues, plastic deformation in the contact area becomes more pronounced, with the peaks of asperities gradually filling into the valleys and forming abrasive particles under repeated friction [29]. With increasing frequency, the number of wear cycles per unit time rises, allowing debris to be promptly expelled from the wear zone. Meanwhile, due to surface softening of the superalloy, the cutting action of debris acting as abrasive particles intensifies. This leads to an increase in surface scratches and deeper grooves on the superalloy, resulting in larger volumes of material being removed from the surface, and thus an increase in wear volume.

### 3.2. Wear Mechanism

#### 3.2.1. Effect of Surface Morphology on Wear Mechanism Under Different Loads

**Figure 8:**
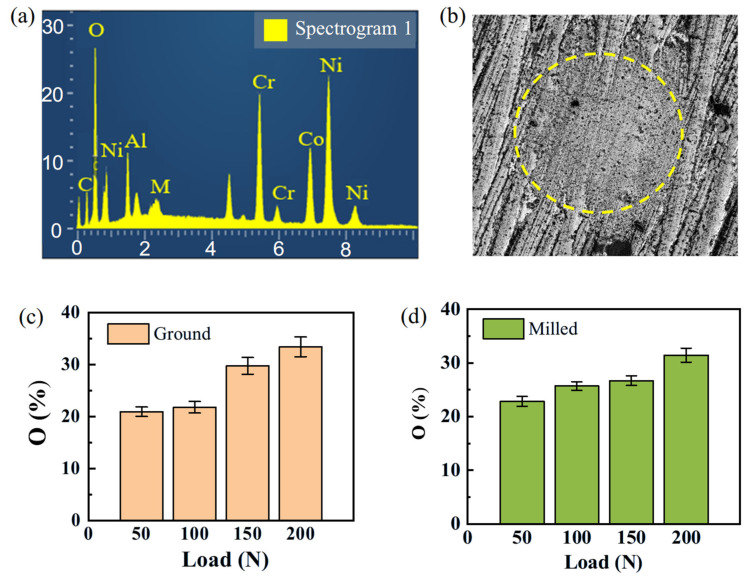
(**a**) EDS quantitative analysis; (**b**) EDS analysis zone; (**c**) oxygen element of ground specimens; (**d**) oxygen element of milled specimens.

The high-temperature testing environment has a significant impact on the fretting wear behavior of superalloy [30,31,32]. EDS is normally used to analyze the weight percentage of oxygen elements [33]. Figure 8a shows the corresponding EDS spectrum of the wear scar. The location of the area where oxygen elements were analyzed is shown by the dotted circle in Figure 8b. The results indicate that when the frequency is held constant, the oxygen content increases with increasing load [34]. Severe oxidative wear occurred on the surfaces of both types of specimen. During the process of oxidative wear, a lubricating oxide film is formed in the wear area. This oxide film has the characteristics of being strong and stable, which can delay the progress of wear and reduce material wear.

**Figure 9:**
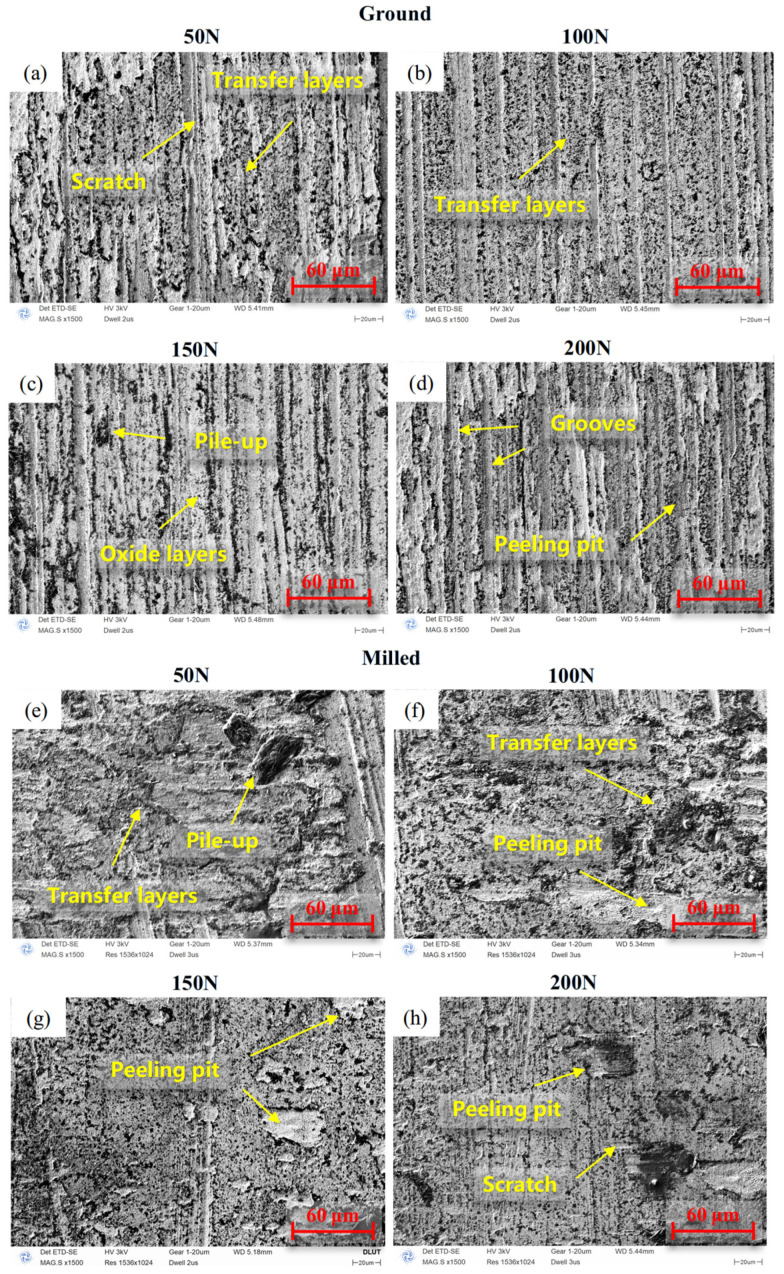
SEM micrographs under different loads of FGH96: (**a**–**d**) ground specimens; (**e**–**h**) milled specimens.

Figure 9 presents the SEM micrographs of ground and milled specimens under different loads. Under low load conditions (50 N and 100 N), due to the formation of significant tangential stresses on the rubbing surfaces, material displacement occurs, resulting in the formation of large-area transfer layers on both types of specimens. Additionally, the ground surface exhibits scratches, indicating that the wear mechanisms are adhesive wear and abrasive wear; the milled surface shows small-area spalling pits, suggesting that the wear mechanisms are adhesive wear and fatigue wear. Under low loads, the dominant wear mechanisms are adhesive wear and abrasive wear. Direct contact between surface asperities induces material transfer and plowing effects, resulting in a relatively high CoF.

Under high load conditions (150 N and 200 N), the ground surface displays deeper plowing grooves. Abrasive debris, subjected to repeated friction and compression by the counter face, can form an oxide film [35], which provides protection and lubrication for the wear area, resulting in wear mechanisms dominated by abrasive wear and oxidative wear; the milled specimens exhibit distinct spalling pits, which are attributed to large-area material removal from the wear surface under higher loads, with the primary wear mechanism being fatigue wear. With the increase in load, the predominant wear mechanisms shift to oxidative wear and fatigue wear. The lubricating effect of oxide layers combined with the spalling of transfer layers collectively contribute to a reduction in the c CoF.

#### 3.2.2. Effect of Surface Morphology on Wear Mechanism Under Different Frequencies

**Figure 10:**
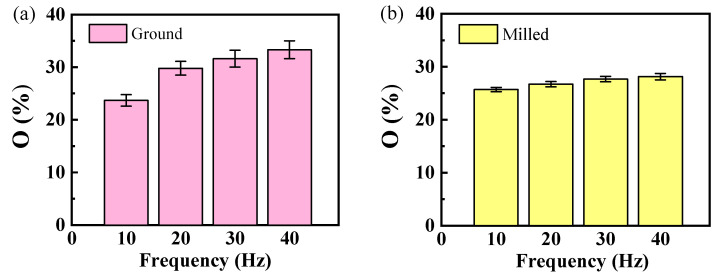
Oxygen percentage at the fretted surface under different frequencies: (**a**) ground specimens, (**b**) milled specimens.

Figure 10 shows the oxygen content on the surfaces of ground and milled specimens after fretting wear under different frequency conditions. The results indicate that when the load is held constant, the oxygen content increases with increasing frequency [36], with the milled specimens showing a relatively smaller influence on the oxygen content. The surface of the FGH96 alloy forms a relatively complete oxide layer. During subsequent friction processes, some of these oxides fill into the grooves generated by abrasive wear, effectively reducing the shedding of surface material and enhancing the wear resistance of the material itself.

**Figure 11:**
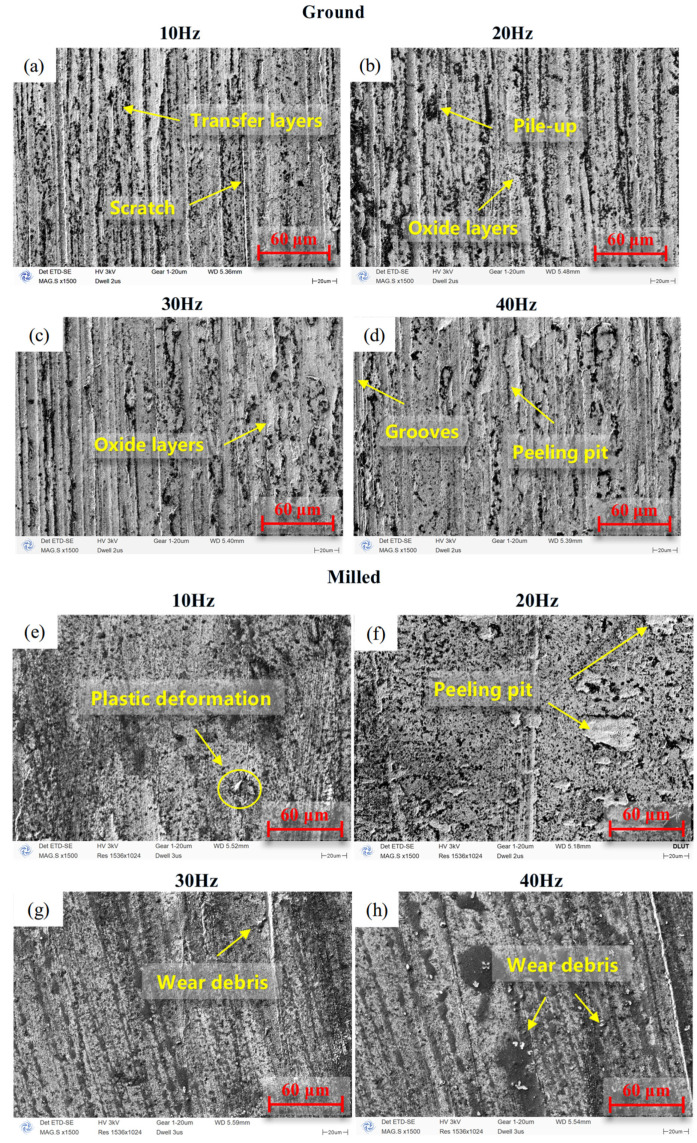
SEM micrographs at the fretted surface under different frequencies: (**a**–**d**) ground specimens, (**e**–**h**) milled specimens.

Figure 11 presents the SEM micrographs of ground and milled specimens under different frequency conditions. Under the low-frequency condition of 10 Hz, the ground surface exhibits a distinct transfer layer as well as scratches, indicating that the abrasive wear and adhesive wear are the main wear mechanisms of ground specimens. The milled specimen shows plastic deformation. Under high-frequency conditions, due to continuous crushing and accumulation, the debris forms a third body with the ability to slow down the degree of fretting wear. The contact mode of the worn area was transformed into three-body contact [37]. Moreover, intense stress concentration caused by repeated friction between the upper and lower specimens eventually leads to material spalling, with wear mechanisms dominated by oxidative wear and fatigue wear. The milled surface displays distinct peeling pits and wear debris, indicating that the wear mechanisms are fatigue wear and abrasive wear.

### 3.3. Surface Morphology Characterization for High-Temperature Fretting Wear

**Figure 12:**
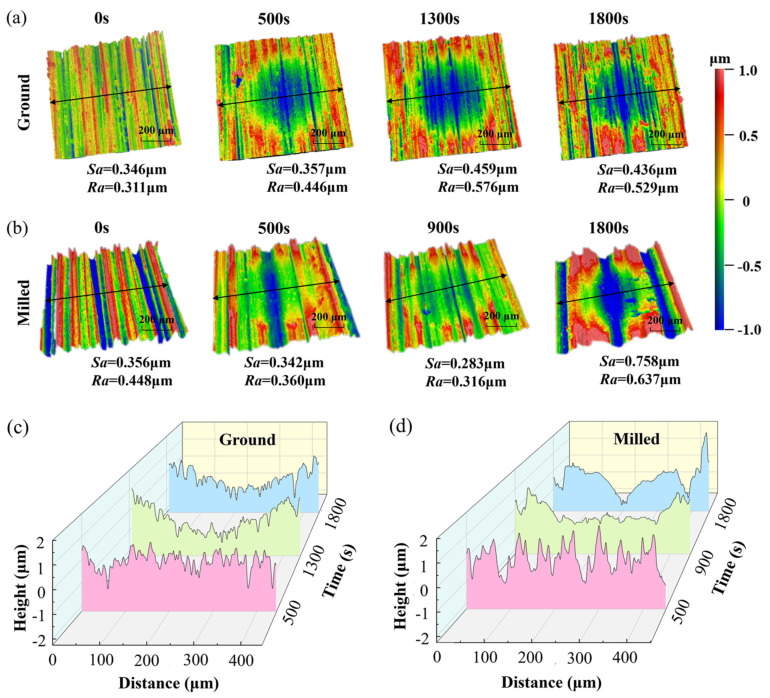
Three-dimensional surface morphologies and line profiles perpendicular of FGH96: (**a**,**c**) ground specimens; (**b**,**d**) milled specimens.

Several surface roughness parameters such as *Ra*, *Rmax*, *Rz* and *Rmr* are normally used to evaluate the surface morphology. In this paper, the average value of Ra was employed as a conventional method for surface morphology. Figure 12 presents the three-dimensional morphology and two-dimensional profiles of the wear zones of ground and milled specimens at different wear stages. The color-bar ranges from 1 μm to −1 μm. The arithmetic mean roughness (*Ra*) was measured at the location perpendicular to the surface machining direction where the wear scar was deepest, while the root mean square (RMS) surface height (*Sa*) was measured within a 600 × 600 μm area containing the wear trace.

As seen in Figure 12a, during the fretting wear process of the ground specimen up to 1300 s, both *Ra* and *Sa* gradually increased, reaching their maximum values at 1300 s. As wear continued, the roughness decreased, with significant debris accumulation observed at the wear edges. As seen in Figure 12b, for the milled surface during the first 500 s of fretting wear, the roughness showed little change, and the wear scars were shallower compared to those on the ground surface. A large amount of wear debris accumulated at the wear edges. At 900 s, the roughness was minimal, with deeper and more uniform wear scars. In the final stage of wear, distinct pits appeared on the surface, resulting in the highest roughness. Figure 12c,d show the cross-sectional profile curves perpendicular to the machining direction of the two specimens at different wear times. For the ground specimen, the profile curve at 1300 s was the roughest, while at 1800 s, it became relatively smoother but still rougher than in the initial state. For the milled specimen, the profile curve at 900 s was the smoothest and flattest, indicating the best surface quality, while at 1800 s, it was the roughest.

**Figure 13:**
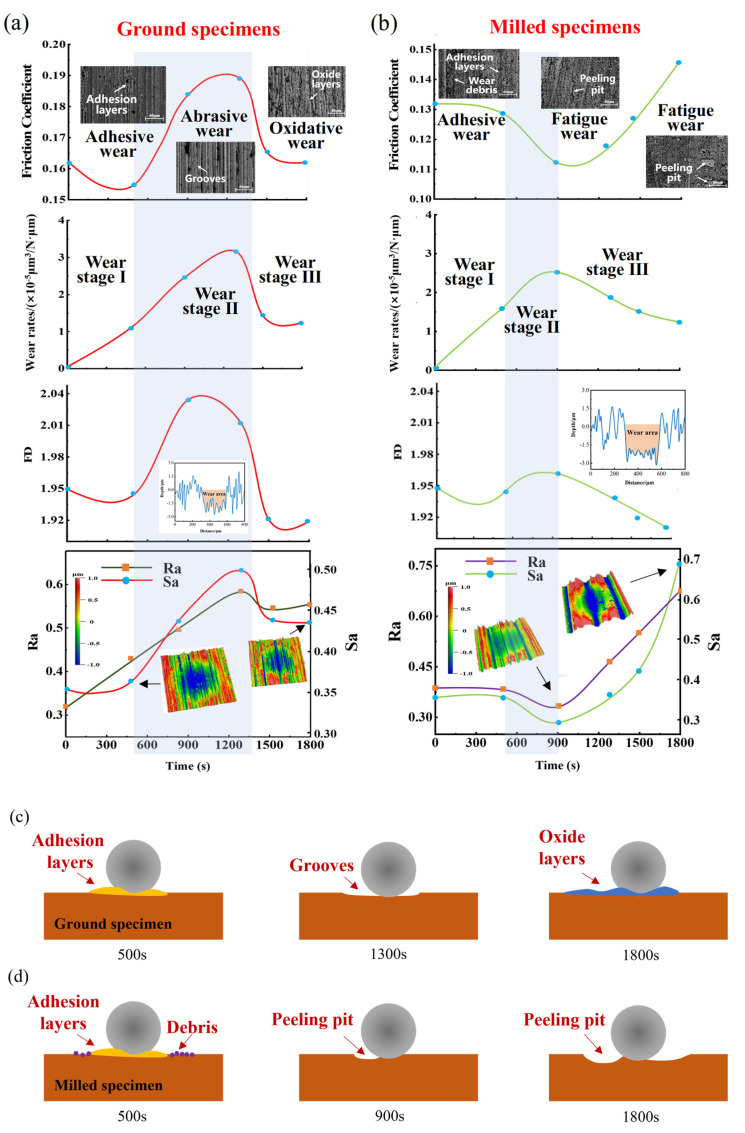
Changes in CoF, wear rate, *FD*, *Ra*, *Sa*, and schematic diagram during the wear process of FGH96: (**a**,**c**) ground specimens; (**b**,**d**) milled specimens.

Figure 13a,b show the variation curves of the CoF, wear rate, fractal dimension (*FD*), *Sa*, and *Ra* for the ground and milled specimens during the wear process, respectively. Figure 13c,d illustrate the fretting wear processes of the ground and milled specimens under the conditions of 150 N load and 20 Hz frequency, respectively.

In the early stage of wear, the ground specimen exhibited a distinct adhesive layer on its surface (Figure 13a,c), while the milled specimen had both an adhesive layer and a small amount of wear debris (Figure 13b,d). The wear mechanism for both specimens was primarily adhesive wear. The formation of the adhesive layer increased surface roughness but reduced surface complexity. Consequently, the CoF and fractal dimension gradually decreased, while the wear rate and roughness parameters increased (as shown in Figure 13a,b).

In the middle stage of wear, the plowing of the ground specimen by the Si_3_N_4_ ball became more severe, resulting in deeper plowing grooves and a more complex surface morphology. The wear mechanism was abrasive wear (Figure 13a,c), leading to increases in the CoF, roughness, fractal dimension, and wear rate (Figure 13a). For the milled specimen, slight fatigue wear caused small-area spalling pits to appear on the surface. The material between the contact surfaces was further expelled from the wear zone, making the surface smoother (Figure 13b,d). Therefore, the CoF and roughness decreased, while the wear rate increased. However, the formation of spalling pits increased surface complexity, resulting in an increase in fractal dimension (Figure 13b).

In the late stage of wear, the plowing grooves on the ground surface disappeared, and the wear mechanism was predominantly oxidative wear. The oxide film mitigated the wear severity and masked some high-frequency information (Figure 13a,c), leading to gradual decreases in surface roughness, fractal dimension, CoF, and wear rate (Figure 13a). For the milled specimen, intensified fatigue wear resulted in more spalling pits on the surface. The CoF and roughness increased. The micro-asperities on the milled surface were more rapidly pressed into the valleys (Figure 13b,d), reducing surface complexity and slowing the wear rate. Therefore, the fractal dimension and wear rate decreased (Figure 13b).

Based on the experimental results, the three roughness parameters of the ground specimen show similar trends to the changes in the CoF and wear rate. In contrast, for the milled specimen, the variation in the CoF is only similar to *Sa* and *Ra*, but is not related to *FD*. The trend in wear rate is similar to *FD* but not to *Sa* and *Ra*. In summary, the roughness parameters *Sa* and *Ra* are strongly correlated with the CoF, while *FD* is strongly correlated with wear rate.

## 4. Conclusions

This study investigated the tribological properties of ground and milled specimens under fretting wear at 700 °C, load ranging from 50 N to 200 N, and fretting frequency ranging from 10 Hz to 40 Hz. The results are summarized as follows:

(1) With the increase in the load and frequency, the CoF for both ground and milled specimens decrease steadily, while the wear volume generally exhibits an increasing trend.

(2) At low frequencies and low loads, wear mechanism is characterized by adhesive wear and abrasive wear for both ground and milled specimens. At high frequencies and high loads, oxidative wear dominates for ground specimens, while fatigue wear dominates for milled specimens.

(3) A novel method to characterize the surface morphology evolution of high-temperature fretted surfaces is proposed by using the *FD* value combined with surface roughness. With the increase in time, the roughness parameters *Sa* and *Ra* were strongly correlated with the CoF, while the *FD* was strongly correlated with wear rate, for both ground and milled specimens.

This study could provide a novel approach to characterizing the evolution of surface morphology during high-temperature fretting wear, and guidance for the determination of machining parameters for the critical aero-engine components.

## Figures and Tables

**Table 1:** Main chemical composition of FGH96 (mass fraction, %).

Element	Cr	Co	W	Mo	Al	Ti	Nb	Zr	C	Ni
wt%	16.20	13.10	1.03	4.08	2.18	3.66	0.71	0.04	0.04	Bal.

**Table 2:** Properties of FGH96.

Materials	Hardness (HV)	σ_0.2_ (MPa)	σ_b_ (MPa)	ε (%)
FGH96	502.4 ± 6.8	1140 ± 48.7	2020 ± 132.0	34.5 ± 7.4

**Table 3:** Experiment conditions of fretting wear.

Influence Factor	Frequency (Hz)	Load (N)	Hertz Contact Pressure (MPa)	Test Duration (s)
Load	20	50	2223	1800
		100	2801	
		150	3207	
		200	3529	
Frequency	10, 20, 30, 40	150	3207	1800
Test duration	20	150	3207	500
				900
				1300
				1800

## Data Availability

The original contributions presented in the study are included in the article, further inquiries can be directed to the corresponding author.
